# Neuraminidase1 Inhibitor Protects Against Doxorubicin-Induced Cardiotoxicity *via* Suppressing Drp1-Dependent Mitophagy

**DOI:** 10.3389/fcell.2021.802502

**Published:** 2021-12-17

**Authors:** Yating Qin, Chao Lv, Xinxin Zhang, Weibin Ruan, Xiangyu Xu, Chen Chen, Xinyun Ji, Li Lu, Xiaomei Guo

**Affiliations:** ^1^ Department of Cardiology, Tongji Hospital, Tongji Medical College, Huazhong University of Science and Technology, Wuhan, China; ^2^ Department of Cardiology, The Second Hospital of Shandong University, Jinan, China; ^3^ Department of Cardiology, The Third People’s Hospital of Hubei Province, Wuhan, China; ^4^ Department of Cardiology, Renmin Hospital of Wuhan University, Wuhan, China

**Keywords:** doxorubicin, cardiotoxicity, neuraminidase1, oseltamivir, dynamin-related protein 1 (Drp1), mitophagy

## Abstract

Anthracyclines, such as doxorubicin (DOX), are among the effective chemotherapeutic drugs for various malignancies. However, their clinical use is limited by irreversible cardiotoxicity. This study sought to determine the role of neuraminidase 1 (NEU1) in DOX-induced cardiomyopathy and the potential cardio-protective effects of NEU1 inhibitor oseltamivir (OSE). Male Sprague–Dawley (SD) rats were randomized into three groups: control, DOX, and DOX + OSE. NEU1 was highly expressed in DOX-treated rat heart tissues compared with the control group, which was suppressed by OSE administration. Rats in the DOX + OSE group showed preserved cardiac function and were protected from DOX-induced cardiomyopathy. The beneficial effects of OSE were associated with the suppression of dynamin-related protein 1 (Drp1)-dependent mitochondrial fission and mitophagy. In detail, the elevated NEU1 in cardiomyocytes triggered by DOX increased the expression of Drp1, which subsequently enhanced mitochondrial fission and PINK1/Parkin pathway-mediated mitophagy, leading to a maladaptive feedback circle towards myocardial apoptosis and cell death. OSE administration selectively inhibited the increased NEU1 in myocardial cells insulted by DOX, followed by reduction of Drp1 expression, inhibition of PINK1 stabilization on mitochondria, and Parkin translocation to mitochondria, thus alleviating excessive mitochondrial fission and mitophagy, alleviating subsequent development of cellular apoptotic process. This work identified NEU1 as a crucial inducer of DOX-induced cardiomyopathy by promoting Drp1-dependent mitochondrial fission and mitophagy, and NEU1 inhibitor showed new indications of cardio-protection against DOX cardiotoxicity.

## 1 Introduction

Doxorubicin (DOX), an extensively prescribed and the most potent chemotherapeutic agent for various malignancies, still remains footstones in oncotherapy combined with emerging targeted drugs. However, its clinical use is limited ascribed to dose-dependent cardiotoxicity, leading to irreversible cardiomyopathy and heart failure ultimately (Swain et al., 2003; Yeh, 2006). Although clinical evaluation makes it possible to detect cardiotoxicity earlier (Thavendiranathan et al., 2014; Vejpongsa and Yeh, 2014; Cardinale et al., 2015), no consensus has been reached on the best way to prevent DOX-induced cardiotoxicity, emphasizing an immediate need for developing novel therapeutic agents.

Accumulating evidence shows that DOX cardiotoxicity is closely linked to mitochondrial damage. Mitochondria are highly dynamic organelles that undergo successive fusion and fission to maintain an appropriate population for mitochondrial quality control. GTPase dynamin-related protein 1 (Drp1) is an essential regulator of mitochondrial fission (Fonseca et al., 2019), and Drp1-mediated mitochondrial fission has been reported to contribute to various cardiovascular diseases, such as diabetic cardiomyopathy, ischemia/reperfusion cardiac injury, and DOX-induced cardiomyopathy (Xia et al., 2017; Zhou et al., 2017; Ding et al., 2018; Lee et al., 2020; Zhuang et al., 2021). Consistently, mitochondrial fission occurs in coordination with mitophagy (Morales et al., 2020), which is a selective form of autophagy that targets elimination of damaged or unfunctional mitochondria. When excessive mitochondrial fission occurs, the number of functional mitochondria is extensively reduced along with an accumulation of mitochondrial fragmentations. Then, the mitophagy process is triggered to digest and clear those structures, attempting to maintain a healthy mitochondrial network (Palikaras et al., 2018). However, upon prolonged stress, mitophagy can also be detrimental to the heart by aggravating mitochondrial damage and accelerating cellular death *via* excessive self-consumption, resulting in cardiac dysfunction (Morales et al., 2020). A recently published study (Catanzaro et al., 2019) identified that Drp1 knockdown could attenuate DOX-induced accelerated mitophagy flux, and Drp1-deficient mice were protected from DOX-induced cardiac damage, strongly confirming the role of Drp1-dependent mitophagy in DOX cardiotoxicity.

Neuraminidases (NEUs), also called sialidases, are a family of glycosidases responsible for the removal of terminal sialic acid from glycoproteins and glycolipids. In mammals, four types of NEUs (NEU1, NEU2, NEU3, and NEU4), encoded by different genes, have been identified according to their distinct enzymatic properties and subcellular localization (Glanz et al., 2019). Among them, NEU1 is the most highly expressed in the heart, involved in several cardiovascular diseases (Zhang et al., 2021). Researchers discovered that NEU1 was highly expressed in the heart of patients with coronary artery disease, and NEU1 knockdown notably protected cardiomyocytes from ischemic injury (Zhang et al., 2018). Overexpression of NEU1 promoted atherosclerosis development and plaque instability by enhancing pro-inflammatory cytokine expression (Sieve et al., 2018). In addition, in ischemia/reperfusion mice model, NEU1 expression and activity were increased in cardiomyocytes as well as in invading monocytic cells, contributing to stronger inflammation and eventually heart failure (Heimerl et al., 2020). What is more, a recently published study identified that NEU1 acted as a crucial driver in cardiac hypertrophy by interplaying with transcriptional factor GATA4 (Chen et al., 2021). However, there are rare researches exploring the relationship between NEU1 and DOX-induced cardiomyopathy so far. In this study, we proposed the hypothesis that NEU1 was a mediator of DOX-induced cardiomyopathy and NEU1 inhibitor could improve DOX-induced cardiac dysfunction through modulating Drp1-dependent mitochondrial fission and mitophagy.

## 2 Materials and Methods

### 2.1 Drugs and Materials

DOX (S1208) was purchased from Selleck, and oseltamivir (HY-17016) was obtained from MedChemExpress (MCE). ELISA kits for NEU1 and N-acetylneuraminic acid (Neu5Ac) were purchased from Jingmei Biotechnology (Jiangsu, China). ELISA kits for creatine kinase isoenzyme-MB (CK-MB) and cardiac troponin T (cTnT) were obtained from Meilian Biotechnology. Commercial assay kits of aspartate aminotransferase (AST), lactate dehydrogenase (LDH), superoxide dismutase (SOD), total antioxidant capacity (T-AOC), reduced glutathione (GSH) activities, and hydrogen peroxide (H_2_O_2_) were purchased from Nanjing Jiancheng Bioengineering Institute (Nanjing, China). Terminal deoxynucleotidyl transferase-mediated dUTP nick end labeling (TUNEL) assay was obtained from Beyotime Biotechnology (Shanghai, China). Cell Mitochondria Isolation Kit was purchased from Beyotime Biotechnology (China). All primary antibodies used in this study are listed in Supplementary Table S1.

### 2.2 Animal Experiment

**FIGURE 1:**
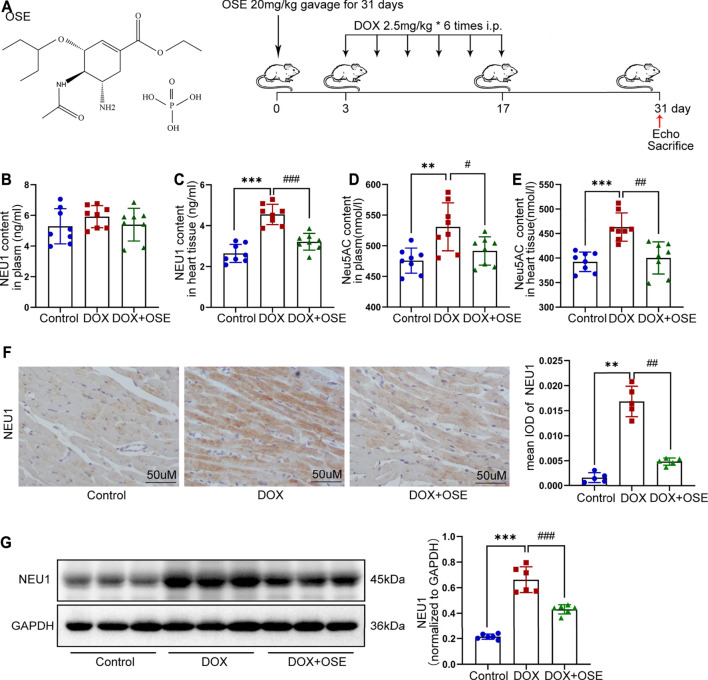
Doxorubicin (DOX) exposure resulted in elevated neuraminidase 1 (NEU1) activity and expression in adult rats. **(A)** The chemical structure of NEU1 inhibitor oseltamivir (OSE) and the schematic diagram of this study. **(B–E)** The content of NEU1 and its regulated downstream metabolite N-acetylneuraminic acid (Neu5AC) in animal plasma or heart tissue measured by commercial ELISA assays (*n* = 8 per group). **(F)** Representative immunohistochemical images of NEU1 expression in rat hearts (*n* = 5 per group, scale bar = 50 μm). **(G)** The expression of NEU1 in rat hearts analyzed by western blot (*n* = 6 per group). (**p < 0.01 vs. control group, ***p < 0.001 vs. control group. #*p* < 0.05 vs. DOX group, ##*p* < 0.01 vs. DOX group, ###*p* < 0.001 vs. DOX group).

Adult male Sprague–Dawley (SD) rats (7 weeks old) were purchased from Huazhong University of Science and Technology in a total number of 36. Rats were housed in sterilized filter top cages in a temperature- and humidity-controlled environment (22 ± 2°C) with a 12-h day–night cycle. After adaptive feeding for a week, rats were randomly assigned into three groups as follows. Rats in the DOX group received intraperitoneal injection of DOX in a cumulative dose of 15 mg/kg within 2 weeks (2.5 mg/kg for six times), mimicking the human therapeutic regimens. Rats in the DOX + OSE group received gavage of OSE (20 mg/kg) dissolved in normal saline beginning from 3 days before the first injection of DOX to the end of the experiment. Rats in the control group were given equal volume of saline for intraperitoneal injection or gavage. The scheme of experiment is shown in Figure 1A. All animal experiments were approved by the Institutional Animal Care and Use Committee of Tongji Medical College, Huazhong University Science and Technology (IACUC number: 2555), which strictly conformed to the National Institutes of Health Guide for the Care and Use of Laboratory Animals.

### 2.3 Echocardiography

After the first injection of DOX for a month, transthoracic echocardiography was conducted to evaluate cardiac function. In brief, rats were tied under anesthetization with 100 mg/kg pentobarbital sodium, and their chest hairs were removed. M-mode images and echocardiographic parameters were obtained when heart rate was about 450 bpm. Ejection fraction (EF) and Fractional shortening (FS) were calculated as previously described.

### 2.4 Histopathology Analysis

Hearts were harvested, weighed, and cut into several pieces, and then, samples were either frozen immediately in liquid nitrogen for further biochemistry detection or fixed in 4% paraformaldehyde for histopathological analysis. For histopathological analysis, after being fixed, dehydrated, and embedded in paraffin blocks, heart samples were cut into thin cross sections at 5 μm. To investigate the pathological morphology of the heart, myocardial fibrosis, and cardiomyocyte size, hematoxylin and eosin (HE) staining, Masson’s trichrome staining, and wheat germ agglutinin (WGA) staining were conducted strictly following manufacturer’s instructions, respectively. All images were obtained by an optical microscope (Olympus, Tokyo, Japan) and analyzed by Image-Pro Plus 6.0 software.

### 2.5 Biochemical Indexes of Myocardial Injury and Oxidative Stress

Cardiac injury parameters and oxidative stress indexes in adult rat serum were measured. Briefly, blood samples were collected and centrifuged at 3,000 rpm for 10 min at 4°C to obtain the serum, which was directly applied for biochemical detection or stored at −80°C until use. The levels of CK-MB and cTnT were measured by ELISA kits. The levels of AST and LDH were detected by commercial assay kits. Then, the levels of oxidative stress indexes including SOD, T-AOC, GSH, and H_2_O_2_ were measured by commercially available assay kits too. All detections were strictly in accordance with the manufacturer’s instructions.

### 2.6 Analysis of the Levels of NEU1 and Neu5AC in Rat Hearts and in Serum

The levels of NEU1 and its downregulated metabolite Neu5AC in adult rat hearts and blood serum were measured using ELISA kits. In brief, left ventricular samples were quickly homogenized in PBS on ice (100 mg/ml), and then, homogenate was obtained for later detection after centrifugation at 1,000 g for 10 min at 4°C following manufacturer’s protocol.

### 2.7 Immunohistochemistry and Immunofluorescence Analyses

Formalin-fixed, paraffin-embedded heart sections were deparaffinized, rehydrated in xylene and gradient ethanol, boiled with an antigen retrieval solution for 20 min, and then incubated with 3% hydrogen peroxide for 30 min after cooling down. Next, heart sections were blocked with 5% goat serum in PBS for 1 h at room temperature before incubation with different primary antibodies overnight at 4°C. For immunohistochemical analysis, sections were incubated with secondary antibody prior to staining with diaminobenzidine then counterstaining with hematoxylin; images were obtained by a light microscope (Olympus). For immunofluorescence analysis, after incubation with secondary antibody and counterstaining with DAPI, images were observed and attained by a fluorescent microscope (Olympus). The positive staining areas were calculated and analyzed by Image-Pro Plus 6.0 software.

### 2.8 Transmission Electron Microscopy Analysis

Hearts were harvested, and left ventricular walls were quickly cut into 1-mm^3^ pieces, which were fixed in 2.5% glutaraldehyde with 0.1 M sodium cacodylate buffer for 2 h at room temperature before being stored at 4°C overnight. Heart samples were washed in 0.1 M sodium cacodylate buffer three times for 30 min, then immersed in 1% osmium tetroxide with 0.1 M sodium cacodylate buffer. After being dehydrated, embedded, and counterstained, ultrathin sections were imaged on a transmission electron microscope (Hitachi-151 HT7800, Japan).

### 2.9 Terminal Deoxynucleotidyl Transferase-Mediated dUTP Nick End Labeling Staining

Paraffin-embedded adult rat heart sections were deparaffinized and rehydrated, then processed for the One-Step TUNEL Apoptosis Assay Detection Kit according to the manufacturer’s protocol. The average apoptotic index was defined as the ratio of the number of TUNEL positively stained nuclei to the number of DAPI-stained nuclei.

### 2.10 Protein Extraction and Immunoblotting

For extraction of the total protein, rat hearts and H9C2 cells were lysed on ice for 15 min in commercial RIPA buffer supplemented with protease inhibitor and phosphatase inhibitor. Lysates were centrifuged at 12,500 rpm for 15 min at 4°C to obtain supernatants. Different from total protein extraction, mitochondrial protein extraction from H9C2 cell was performed using a commercial Cell Mitochondria Isolation Kit. In brief, cells were digested by trypsin, collected in PBS, and centrifuged at 100 g for 5 min at room temperature to obtain sediments. Mitochondria isolation reagent was added, and cells were homogenized several times on ice until the percentage of trypan blue positively stained cells was approximately 50%. Cells were then centrifuged at 600 g for 10 min at 4°C to obtain supernatants, which were centrifuged again at 11,000 g for 10 min at 4°C. Then, the supernatants contained cytosolic protein while the sediments were purified mitochondria. Next, supernatants were centrifuged at 12,500 rpm for 15 min at 4°C to obtain supernatants, which were purified cytosol protein. The purified mitochondria were lysed on ice for 30 min in mitochondria lysis buffer, followed by centrifugation at 12,500 rpm for 15 min at 4°C to obtain supernatants, which were purified mitochondrial protein. Protein concentration was determined by using a bicinchoninic acid assay. Denatured proteins were separated by sodium dodecyl sulfate-polyacrylamide gel electrophoresis and transferred onto polyvinylidene difluoride membranes. Membranes were blocked with 5% bovine serum albumin (BSA) for 1 h at room temperature and subsequently incubated with different primary antibodies at 4°C overnight. After incubation with appropriate peroxidase-conjugated secondary antibodies for 1 h at room temperature, signals were detected with enhanced chemiluminescence.

### 2.11 Cell Culture and Treatment

H9C2 cell line was purchased from the American Type Culture Collection (ATCC, United States) and was cultured in Dulbecco’s modified Eagle’s medium, supplemented with 10% fetal bovine serum and 100 U/ml penicillin/streptomycin, at 37°C with 5% CO_2_. To investigate the effect of DOX on H9C2 cell line in different times, cells cultured at about 80% confluence were treated with 1 μM DOX for 3, 6, and 12 h, respectively. To study the effect of OSE against DOX-induced myocardial injury, cells were treated as follows: cells in the control group were treated with culture medium only; cells in the DOX group were treated with 1 μM DOX for 12 h; and cells in the DOX + OSE group were pretreated with 2.5, 5, and 10 μM OSE for 2 h, respectively, followed by incubation with 1 μM DOX for 12 h.

### 2.12 Statistical Analysis

All data were statistically analyzed using Prism software (GraphPad software 8.0) and shown as Mean ± SEM. Log-rank test was used for survival analysis. *p* values were calculated with one-way ANOVA or Student’s *t*-test as appropriate. *p* values <0.05 were considered statistically significant.

## 3 Results

### 3.1 Elevated NEU1 Content and Expression in Adult Rats After DOX Exposure

The level of NEU1 in blood serum or left ventricular tissue of adult rats among the three groups was analyzed using a commercial ELISA assay. The level of NEU1 in blood serum tended to be higher in the DOX group in comparison with the other two groups (control vs. DOX: 5.29 ± 1.14 vs. 5.91 ± 0.72 *p* = 0.21; DOX vs. DOX + OSE: 5.91 ± 0.72 vs. 5.39 ± 1.06 *p* = 0.27) (Figure 1B). As regards to the level of NEU1 in myocardial tissue, a significant increase was observed in the DOX group when compared with the control group (*p* < 0.05), while the increase was dramatically restrained by co-treatment with OSE (*p* < 0.05) (Figure 1C). Moreover, DOX exposure resulted in an elevated level of Neu5AC, a kind of metabolite whose generation was predominantly regulated by NEU1, in both blood serum and myocardial tissue compared with the control group, and the elevated level of Neu5AC was significantly attenuated post-OSE treatment (Figure 1D, E). What is more, by immunohistochemical staining, it was revealed that NEU1 expression was dramatically increased in myocardial tissues of DOX-treated rats in comparison with the control group, which was significantly attenuated by OSE co-treatment (Figure 1F). Western blot analysis revealed that the protein expression of NEU1 in left ventricular tissues was in complete accordance with the results of the immunohistochemical staining (Figure 1G). These data indicated that DOX exposure was capable of triggering abnormal activation of NEU1 in adult rat myocardial tissues but not in blood serum, followed by increasing generation of regulated downstream metabolite Neu5AC; however, the abnormally activated processes were substantially prohibited by OSE co-treatment.

### 3.2 NEU1 Inhibitor Improved DOX-Induced Cardiac Dysfunction in Rats

**FIGURE 2:**
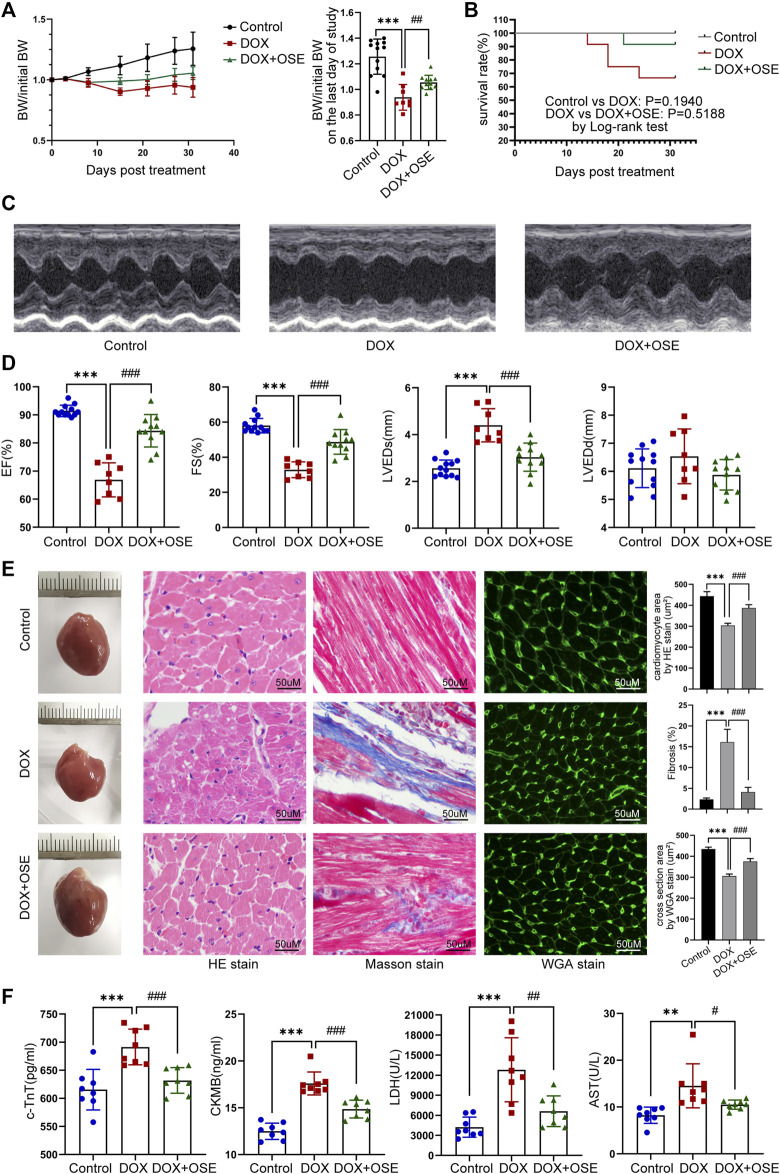
NEU1 inhibitor improved DOX-induced cardiac dysfunction in rats. **(A)** The tendency of body weight change of rats in three groups (*n* = 12 per group). **(B)** The survival curve of rats in different groups. **(C)** Representative images of M-mode echocardiography in different rat groups at the endpoint of the study. **(D)** Statistical analyses of echocardiographic parameters including ejection fraction (EF), fractional shortening (FS), left ventricular end-systolic dimension (LVEDs), and left ventricular end-diastolic dimension (LVEDD) of rats in the three groups at the end of the study. **(E)** Representative images of hematoxylin and eosin (HE) staining, Masson’s trichrome staining, and wheat germ agglutinin (WGA) staining of heart tissues in the three different groups, which display the extent of cardiac atrophy, myocardial fibrosis, and cell sizes, respectively (scale bar = 50 μm). **(F)** Concentrations of cardiac injury markers including cardiac troponin T (cTnT), creatine kinase isoenzyme-MB (CK-MB), lactate dehydrogenase (LDH), and aspartate aminotransferase (AST) in plasma of rats in different groups. (**p < 0.01 vs. control group, ***p < 0.001 vs. control group. #*p* < 0.05 vs. DOX group, ##*p* < 0.01 vs. DOX group, ###*p* < 0.001 vs. DOX group).

Based on the hypothesis that abnormally activated NEU1 activity in rat hearts was one of major contributors in DOX-induced cardiac dysfunction, we next investigated whether NEU1 inhibitor OSE could play a protective role against DOX-induced cardiotoxicity. The ratio of body weight to initial body weight (BW/initial BW) analysis showed that rats in the DOX group had a gradually decreasing body weight post-DOX exposure, while NEU1 inhibitor OSE tended to prevent the body weight loss induced by DOX (Figure 2A). A survival rate analysis depicted no significant differences among the three groups, indicating that the dose of DOX we used was well tolerated by adult rats (Figure 2B). Then, the ratios of heart weight (HW), lung weight (lung W), or liver weight (liver W) to tibial length (TL) among the three groups were assessed. A significant decrease of HW/TL ratio was observed in the DOX group in comparison with the control group, which was prevented by OSE co-treatment (Supplementary Figure S1A). No significant difference in lung W/TL ratio or liver W/TL ratio was observed among the three groups (Supplementary Figure S1B, C), indicating the absence of lung and liver congestion in these rats, reminiscent of a subclinical myocardial dysfunction, which was observed in chronic DOX-related cardiotoxicity of the clinical practice.

Echocardiographic evaluation at 4 weeks after the first DOX intraperitoneal injection revealed that DOX induced a significant reduction of cardiac contractility (Figure 2C), manifested by reduced left ventricular ejection fraction (LVEF) and left ventricular fraction shortening (LVFS), compared with the control group, and the reduction was substantially prevented by OSE treatment (Figure 2D). A significant increase of left ventricular end-systolic dimension (LVEDS) but not left ventricular end-diastolic dimension (LVEDD) was observed in DOX-treated rats compared with control rats, and the increase was prevented by OSE co-treatment (Figure 2D). Moreover, HE staining, Masson’s trichrome staining, and WGA staining uncovered cardiac atrophy, increased fibrosis, and decreased cell sizes in the DOX group hearts, respectively, which were attenuated by OSE treatment (Figure 2E). Then, DOX exposure significantly increased the concentrations of cTnT, CK-MB, LDH, and AST in circulation compared the control group, which were alleviated by OSE treatment (Figure 2F). The results above strongly confirmed the cardio-protective effects of OSE against DOX-induced cardiomyopathy.

### 3.3 NEU1 Inhibitor Modulated Autophagy, Mitochondrial Fission, and Mitophagy in DOX-Treated Rat Hearts

**FIGURE 3:**
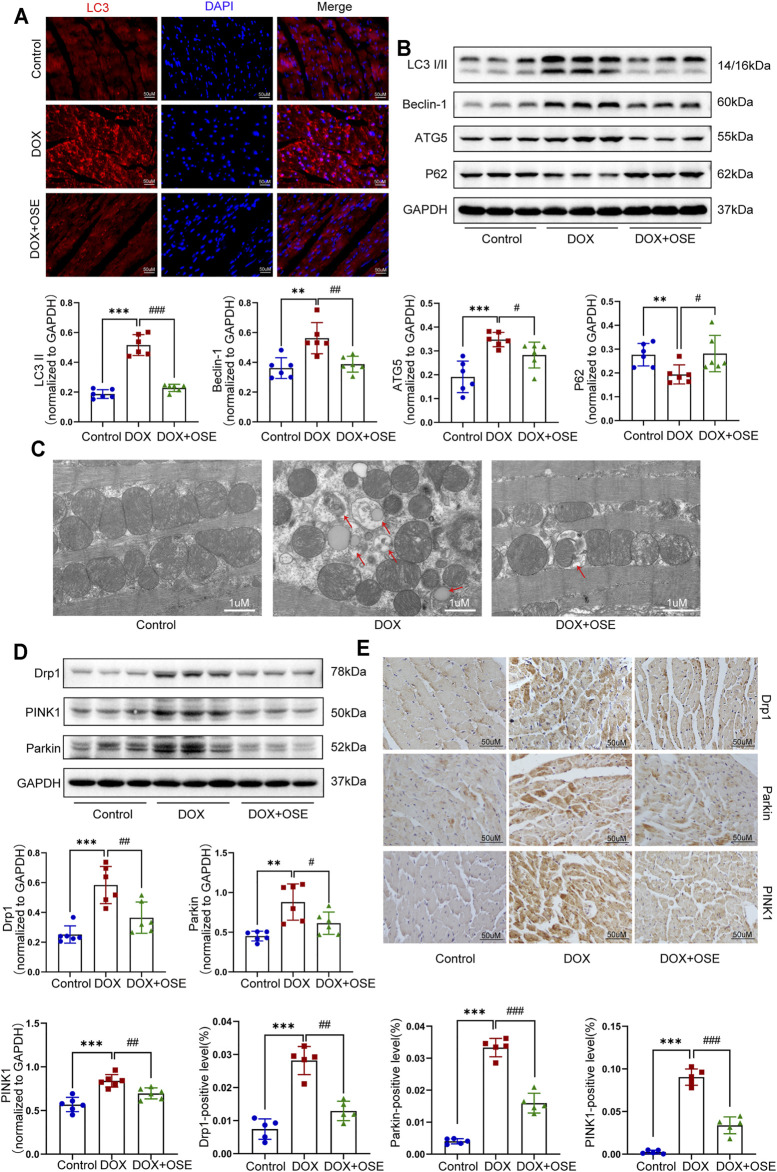
NEU1 inhibitor modulated autophagy, mitochondrial fission, and mitophagy in DOX-treated rat hearts. **(A)** Representative images of LC3II expression in heart tissues of the three groups (scale bar = 50 μm). **(B)** The expression levels of LC3I/II, ATG5, Beclin 1, and P62 in heart tissues of the different groups by western blot (*n* = 6 per group). **(C)** Representative electron microscopy images of heart tissues in the three groups; the red arrows direct autophagic vacuoles containing cargos (scale bar = 1 μm). **(D)** The expression levels of dynamin-related protein 1 (Drp1), PINK1, and Parkin in heart tissues of the three groups by western blot (*n* = 6 per group). **(E)** Representative immunohistochemical images of Drp1, PINK1, and Parkin expressions in heart tissues of the three groups (*n* = 5 per group, scale bar = 50 μm). (**p < 0.01 vs. control group, ***p < 0.001 vs. control group. #*p* < 0.05 vs. DOX group, ##*p* < 0.01 vs. DOX group, ###*p* < 0.001 vs. DOX group).

Emerging evidence has demonstrated that dysregulation of autophagy in myocardium is critically involved in pathophysiologic processes in cardiovascular disease including DOX cardiotoxicity. The possible link between NEU1 and autophagy was thus investigated in DOX-treated rats. The levels of LC3-II, converting from LC3-I and serving as a specific hallmark of autophagy, were significantly increased in DOX-treated rat hearts by immunofluorescence staining and western blot analysis (Figure 3A, B). In contrast, the LC3-II accumulation was dramatically lower in the OSE co-treatment group hearts. Moreover, Beclin 1 and ATG5 are essential components for autophagosome initiation and formation, and we found that, in comparison with the control group, DOX exposure induced much higher protein expressions of these genes, which were effectively suppressed by OSE co-treatment (Figure 3B). In addition, p62/SQSTM1, another protein marker of autophagy, and western blot analysis showed that the protein expression of p62 was much lower in DOX-treated rat hearts than in the control group, while OSE co-treatment significantly suppressed the effect of DOX on P62 expression (Figure 3B). The results above indicate that NEU1 inhibitor OSE may act as a regulator for improving autophagy dysregulation in DOX-induced cardiotoxicity.

Dysregulation of autophagy in myocardium is capable of inducing organelle impairment, especially the mitochondria. Results of transmission electron microscopy revealed an elevated proportion of smaller, fatter, and disorganized mitochondria as well as an increasing number of autophagic vacuoles containing cytosolic cargos including damaged mitochondria in DOX-treated rat hearts compared with the control group (Figure 3C), indicating the initiation of mitochondrial autophagy, which was also called mitophagy. However, OSE co-treatment markedly improved the disorders, including maintaining mitochondrial morphology and reducing autophagosome formation. In mammalian cells, mitophagy occurs in coordination with mitochondrial fission. In parallel with the observation from transmission electron microscopy, western blot analysis and immunohistochemical staining both showed that the expression of Drp1, a key protein controlling mitochondrial fission in mammalian cells, was significantly increased in DOX-treated rat hearts compared with the control group, which was attenuated by OSE co-treatment (Figure 3D, E). In addition, our findings revealed that mitophagy-related critical proteins including PINK1 and Parkin were dramatically increased in DOX-treated rat hearts compared with the control group, which were reduced by OSE co-treatment (Figure 3D, E), indicating that OSE played a protective role against DOX-induced abnormal mitochondrial fission and mitophagy in adult rat hearts.

### 3.4 NEU1 Inhibitor Effectively Prevented Apoptosis in DOX-Treated Rat Hearts

**FIGURE 4:**
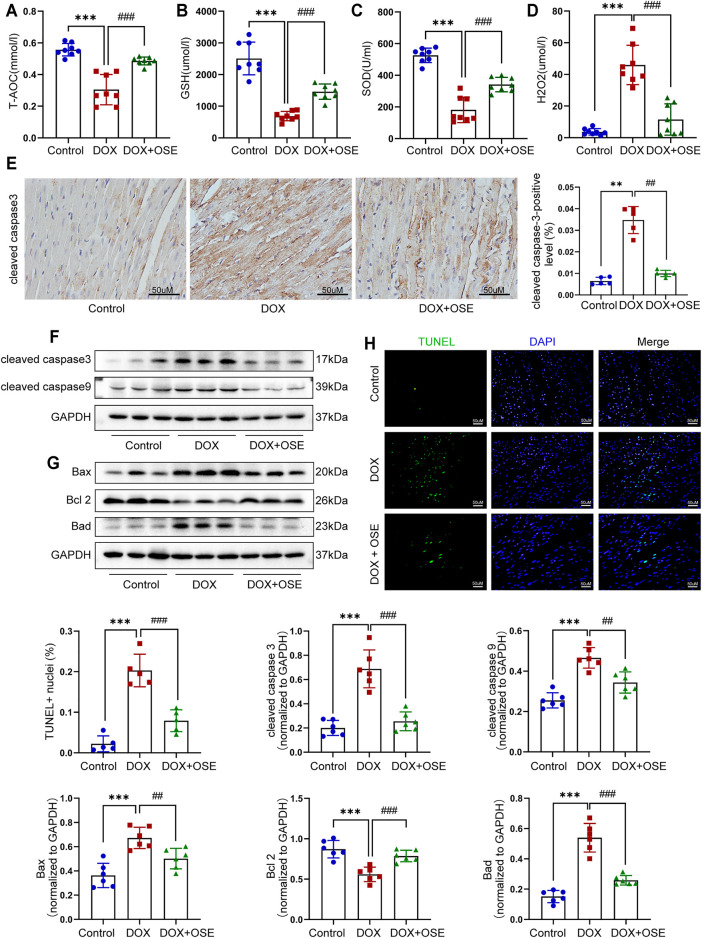
NEU1 inhibitor effectively prevented apoptosis in DOX-treated rat hearts. The concentrations of endogenous antioxidants including total antioxidant capacity (T-AOC) **(A)**, glutathione (GSH) **(B)**, superoxide dismutase (SOD) **(C)**, and endogenous oxidative factor hydrogen peroxide (H_2_O_2_) **(D)** in blood serum measured by commercial assays. **(E)** Representative immunohistochemical images of cleaved caspase-3 abundance in heart tissues of the three groups (*n* = 5 per group, scale bar = 50 μm). The expression levels of cleaved caspase-3 and cleaved caspase-9 **(F)** and Bax, Bcl-2, and Bad **(G)** in heart tissues of the three groups by western blot (*n* = 6 per group). **(H)** Terminal deoxynucleotidyl transferase-mediated dUTP nick end labeling (TUNEL) staining of heart tissues in three groups; positive nuclei were stained in green while nuclei were stained in blue (*n* = 5 per group, scale bar = 50 μm). (**p < 0.01 vs. control group, ***p < 0.001 vs. control group. ##*p* < 0.01 vs. DOX group, ###*p* < 0.001 vs. DOX group).

Abnormal mitochondrial fission and enhanced mitophagy make it hard to maintain a healthy mitochondrial population. Damaged mitochondria not only generate a great deal of reactive oxygen species (ROS) but also have a greater propensity to trigger apoptosis. As shown in Figure 4, DOX treatment significantly reduced the concentrations of endogenous antioxidants including SOD, GSH, and T-AOC in blood serum, whereas it dramatically increased the concentrations of endogenous oxidative factors like H_2_O_2_ compared with the control group, which at least partly indicated that DOX exposure triggered oxidative damage in adult rats, and OSE co-treatment could attenuate the adverse process (Figure 4A–D).

In addition, our data showed that the cleaved caspase-3, a critical marker protein of apoptosis, was more abundant in DOX-treated rat hearts than in the control group, which tended to be less abundant after OSE co-treatment, as evidenced by immunohistochemical staining (Figure 4E) and western blot analysis (Figure 4F). Then, our data demonstrated that DOX exposure resulted in the upregulation of cleaved caspase-9, while OSE co-treatment significantly reversed this phenomenon (Figure 4F). B-cell lymphoma 2 (Bcl-2) family proteins, which are located in the outer membrane of mitochondria, are known regulators of mitochondrial-initiated apoptotic events (Dong et al., 2019). Western blot analysis revealed that DOX treatment significantly increased the expression levels of Bax and Bad but decreased Bcl-2 level in adult rat hearts when compared with the control group, which was suppressed by OSE co-treatment (Figure 4G). TUNEL staining assay also revealed that myocardial sections in the DOX group had much more amounts of apoptotic cardiomyocytes than in the control group, which tended to be less in the OSE co-treatment group (Figure 4H). The results above indicated that the cardio-protective role of OSE against DOX cardiotoxicity was at least partly through inhibiting mitochondrial-mediated myocardial apoptosis.

### 3.5 DOX Induced the Upregulation of NEU1 and Dysfunction of Autophagy and Mitophagy in H9C2 Cells in a Time-Dependent Manner

**FIGURE 5:**
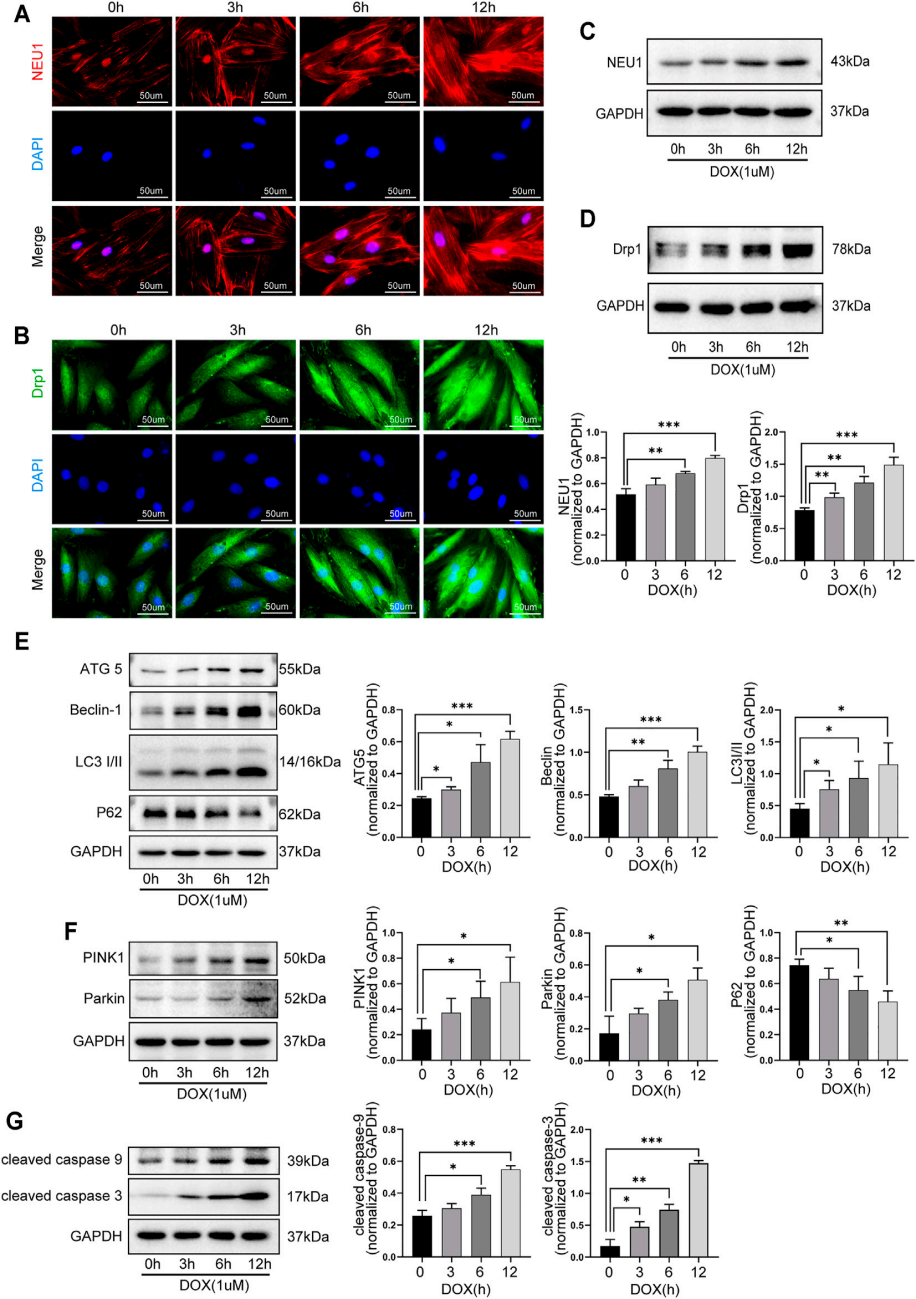
DOX induced the upregulation of NEU1 and dysfunction of autophagy and mitophagy in H9C2 cells in a time-dependent manner. Representative immunofluorescent images of NEU1 **(A)** and Drp1 **(B)** expressions in H9C2 cells that suffered from DOX exposure for 0, 3, 6, and 12 h. Western blot analysis showed that prolonged DOX exposure gradually increased expressions of NEU1 **(C)** and Dpr1 **(D)** in H9C2 cells. **(E)** Prolonged DOX exposure increased the expressions of autophagic markers including ATG5, Beclin 1, and LC3I/II, while it decreased the expression of P62 in H9C2 cells. **(F)** Prolonged DOX exposure enhanced the expressions of mitophagy-related markers like PINK1 and Parkin in H9C2 cells. **(G)** Prolonged DOX exposure activated apoptotic markers including caspase-3 and caspase-9 in H9C2 cells. (*p < 0.05, **p < 0.01, ***p < 0.001).

The role of NEU1 and its possible relationship with autophagy and mitophagy in DOX-induced cardiotoxicity were further explored in H9C2 cells. As shown in Figure 5, the levels of NEU1 and Drp1 in H9C2 cells were gradually increased after DOX exposure for 0, 3, 6, and 12 h, as evidenced by immunofluorescence (Figure 5A, B) and western blot analysis (Figure 5C, D), indicating that prolonged DOX insult led to the upregulation of NEU1 as well as the increase of mitochondrial fission in H9C2 cells in a time-dependent manner. Our results revealed that DOX insult increased autophagosome formation in H9C2 cells, as evidenced by the gradually increased protein levels of ATG5, Beclin 1, and LC3I/II (Figure 5E); meanwhile, DOX exposure promoted the fusion of autophagosome with lysosome in H9C2 cells, as evidenced by the gradually decreased protein level of P62 (Figure 5E). Prolonged DOX exposure seemed to enhance mitophagy as well, as evidenced by gradually increased protein levels of PINK1 and Parkin (Figure 5F), two critical molecules in the canonical pathway of mitophagy. In addition, the levels of cleaved caspase-9 and cleaved caspase-3 were also observed to increase in a time-dependent manner in H9C2 cells after DOX exposure (Figure 5G). These findings strongly suggested that DOX insult was able to upregulate NEU1 expression, enhance mitochondrial fission, and mitophagy in H9C2 cells, followed by myocardial apoptosis.

### 3.6 OSE and Drp1 Inhibitor Mdivi-1 Effectively Blunted Excessive Mitochondrial Fission and Mitophagy in DOX-Exposed H9C2 Cells

**FIGURE 6:**
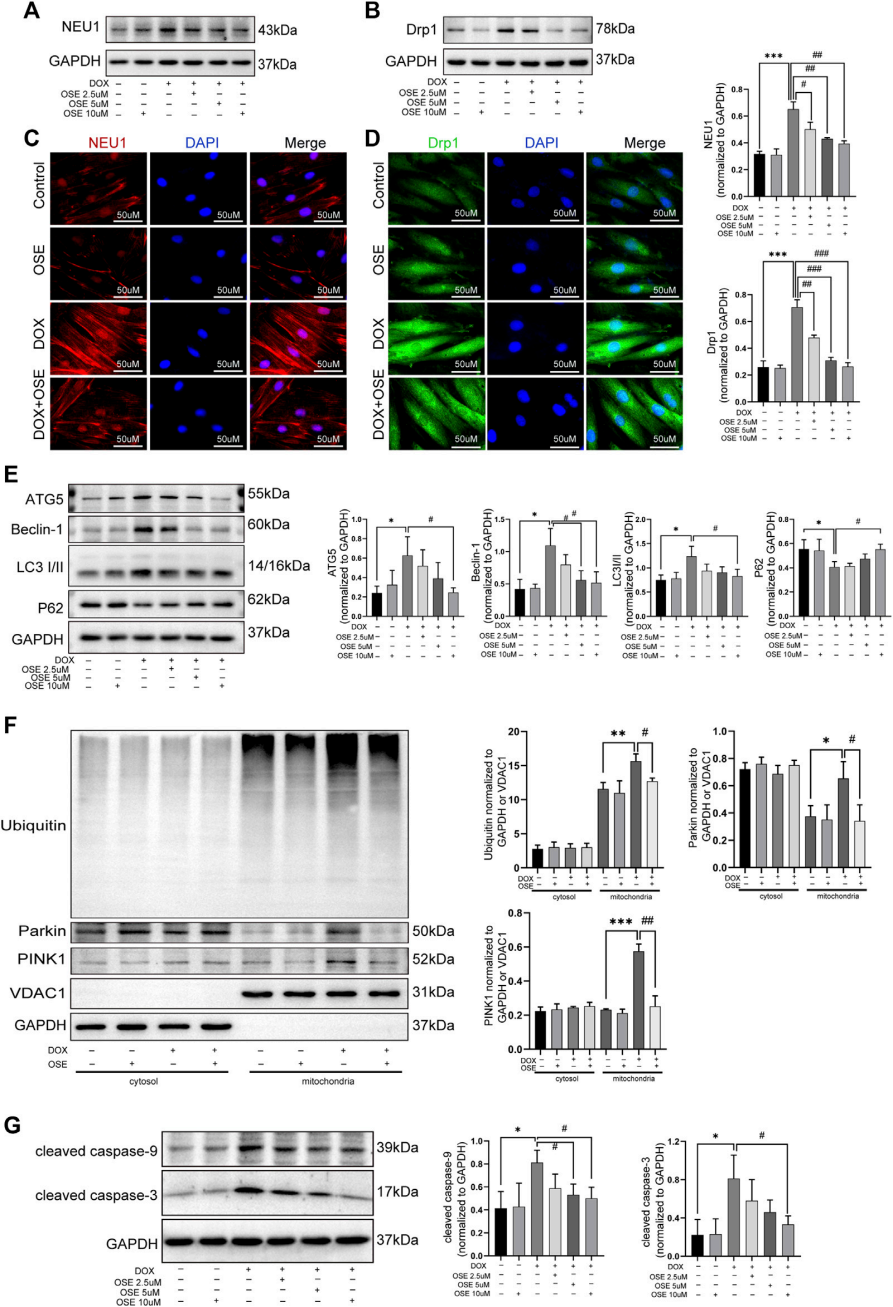
OSE effectively blunted excessive mitochondrial fission and mitophagy in DOX-exposed H9C2 cells. **(A)** OSE suppressed the elevated NEU1 expression induced by DOX in H9C2 cells in a dose-dependent manner. **(B)** OSE suppressed the elevated Drp1 expression induced by DOX in H9C2 cells in a dose-dependent manner. Representative immunofluorescent images of NEU1 expression **(C)** or Drp1 expression **(D)** after OSE treatment in DOX-induced H9C2 cells. **(E)** Western blot analysis revealed that OSE suppressed the excessive autophagy activation induced by DOX in H9C2 cells in a dose-dependent manner. **(F)** Western blot analysis showed that OSE attenuated mitophagy activity in DOX-treated H9C2 cells in a dose-dependent manner through inhibiting PINK1 accumulation, Parkin recruitment, and ubiquitination on mitochondria. **(G)** OSE suppressed apoptotic activity in DOX-treated H9C2 cells in a dose-dependent manner. (*p < 0.05 vs. control group, **p < 0.01 vs. control group, ***p < 0.001 vs. control group. ##*p* < 0.01 vs. DOX group, ###*p* < 0.001 vs. DOX group).

Supposing that the increased mitochondrial fission and mitophagy in DOX-exposed H9C2 cells were ascribed to the upregulation of NEU1, further studies were conducted to investigate whether the NEU1 inhibitor OSE showed protective effects against DOX-induced excessive mitochondrial fission and mitophagy in H9C2 cells. Firstly, western blot analysis revealed that OSE treatment significantly decreased the high expression of NEU1 in DOX-treated H9C2 in a dose-dependent manner (Figure 6A), and the most effective dose of OSE was 10 μM, which was used in the later study. Interestingly, the elevated expression of Drp1 caused by DOX was also decreased consistent with NEU1 after OSE treatment (Figure 6B). The results above were further evidenced by immunofluorescence assays (Figure 6C, D), indicating that the upregulation of Drp1 in DOX-exposed H9C2 cells was ascribed to the elevation of NEU1. What is more, the enhancement of autophagy after DOX insult was suppressed in a dose-dependent manner after OSE treatment, as evidenced by decreased expressions of ATG5, Beclin 1, and LC3II and increased level of P62 (Figure 6E).

Then, we explored the effects of OSE on enhanced mitophagy activity in DOX-treated H9C2 cells after extraction and segregation of mitochondrial protein and cytoplasmic protein. Western blot analysis revealed that the level of PINK1, an inducer of mitophagy, was increased in DOX-treated mitochondria (Figure 6F), confirming that damaged mitochondria elicited by DOX was deprived of degradation of imported PINK1. Accumulated PINK1 on mitochondria could recruit E3 ubiquitin ligase Parkin from cytosol to mitochondria, which subsequently led to the ubiquitination of mitochondrial substrates. This modification got damaged mitochondria ready for being autophagic removal. In keeping with the increased level of PINK1 on mitochondria, the increased translocation of Parkin from cytosol to mitochondria as well as the increased ubiquitination of mitochondria after DOX exposure were evidenced by western blot analysis (Figure 6F), suggesting that DOX treatment enhanced the activity of mitophagy in H9C2 cells. However, the enhanced mitophagy was notably suppressed by the NEU1 inhibitor OSE, as evidenced by less abundant PINK1 accumulation, reduced Parkin recruitment, and decreased ubiquitination on mitochondria after OSE administration (Figure 6F). In addition, we further observed that OSE suppressed the processes of mitochondrial fission and mitophagy, accompanied by the inhibition of apoptotic activities in DOX-treated H9C2 cells (Figure 6G).

**FIGURE 7:**
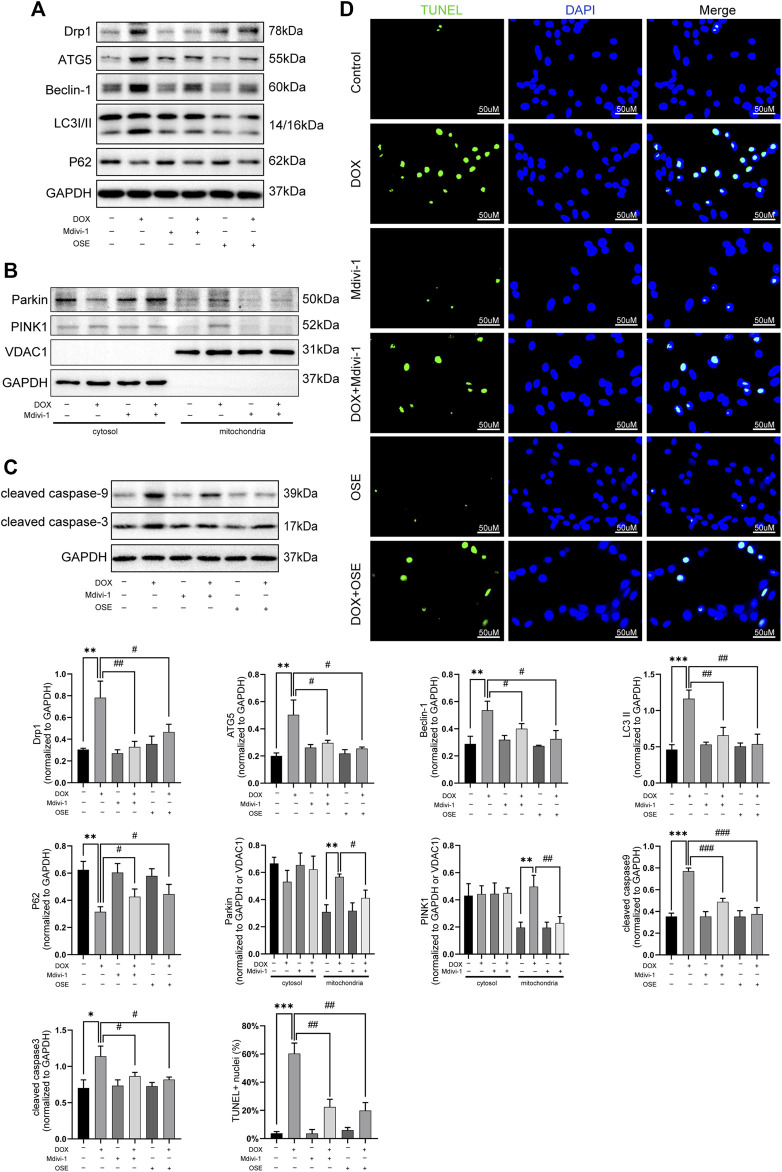
Drp1 inhibitor Mdivi-1 showed similar effects with OSE in DOX-exposed H9C2 cells. **(A)** Mdivi-1 decreased the elevated Drp1 expression and suppressed the enhanced autophagic activity in DOX-treated H9C2 cells, similarly like OSE. **(B)** Mdivi-1 reduced PINK1 accumulation on mitochondria and prevent Parkin translocation from cytosol to mitochondria in DOX-induced H9C2 cells. **(C)** Consistent with the effects of OSE, Mdivi-1 suppressed apoptotic activity in DOX-induced H9C2 cells. **(D)** Representative TUNEL staining images showing that Mdivi-1 suppressed the enhanced apoptosis in DOX-induced H9C2 cells, similarly like OSE.

Last but not the least, the role of Drp1 on enhanced mitophagy was further explored in DOX-treated H9C2 cells. As depicted in Figure 7, Drp1 inhibitor Mdivi-1 not only blunted the increased expression of Drp1 but also suppressed autophagic activities by decreasing the expressions of ATG5, Beclin 1, and LC3II and increasing the expression of P62 (Figure 7A). Consistent with the effects of OSE, Mdivi-1 could also reduce the abundance of PINK1 on mitochondria and prevent the translocation of Parkin from cytosol to mitochondria (Figure 7B). Moreover, the increased apoptosis activity caused by DOX was significantly suppressed by Mdivi-1, as evidenced by the results of TUNEL staining and western blot (Figure 7C, D).

## 4 Discussion

**FIGURE 8:**
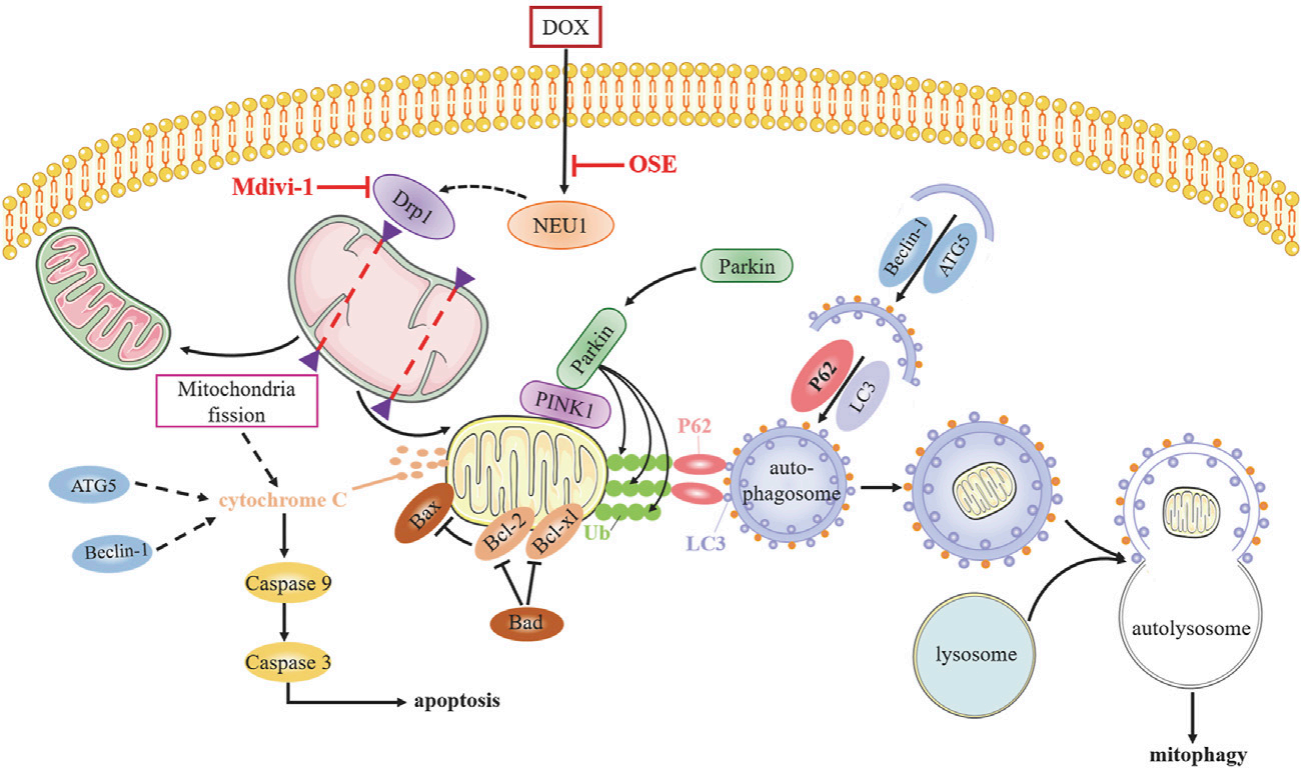
The potential molecular mechanisms of OSE against DOX-induced cardiotoxicity.

Although numerous studies have sought to elucidate the mechanism of anthracycline-induced cardiotoxicity for decades, specific determinants are not yet fully clarified (Renu et al., 2018). The generation of excess ROS and topoisomerase (Top) 2β are most widely accepted contributors facilitating DOX cardiotoxicity progression (Zhang et al., 2012; Rochette et al., 2015); however, neither antioxidants nor iron chelation could completely prevent dilated cardiomyopathy development (Hasinoff et al., 2003; Chow et al., 2015; Ambrosone et al., 2020), indicating multifactorial pathogenic processes responsible for DOX cardiotoxicity. Whether NEU1, a recently identified inducer in various cardiovascular diseases, is involved in DOX cardiotoxicity remains unknown until now. In this study, we uncovered that NEU1 acted as a critical driver of DOX cardiotoxicity, and NEU1 inhibitor had potentials to effectively improve cardiac dysfunction in DOX-induced cardiomyopathy by suppressing Drp1-mediated mitochondrial fission and mitophagy (Figure 8).

The role of NEU1 has been studied in different cardiovascular diseases, including atherosclerosis, ischemic/reperfusion injury, cardiac hypertrophy, and heart failure (Heimerl et al., 2020; Chen et al., 2021), (Gökmen et al., 2000; Süer Gökmen et al., 2006; Yang et al., 2012; Gayral et al., 2014; White et al., 2018). In this study, we observed the elevated content as well as the increased protein expression of NEU1 in the hearts of DOX-treated rats, whereas no significant difference in NEU1 level in the plasma of DOX-treated rats was observed when compared with the control group, strongly suggesting that DOX-induced abnormal activation of NEU1 was in the heart and not in blood. We assumed that this may be ascribed to the distribution and subcellular location of NEU1, since NEU1 is highly expressed in the heart (Pshezhetsky et al., 1997) and predominantly distributed in the cell membrane, cytoplasmic vesicles, and lysosomes in mammals (Igdoura et al., 1998). Similar results were also observed in the mice myocardial ischemic model by Zhang et al. (2018). *In vitro* studies further discovered that the expression of NEU1 was increased in a time-dependent manner under DOX exposure in H9C2 cells. Interestingly, NEU1 inhibitor treatment significantly reduced cardiac injury and notably improved cardiac dysfunction in DOX-treated rats, manifested by decreased level of plasma concentrations of cTnT, CKMB, LDH, and AST and improved left ventricular function. Therefore, we conducted advanced studies to investigate the role of NEU1 in DOX-exposed rat hearts and discovered that increased NEU1 content was associated with Drp1-mediated excessive mitochondrial fission and mitophagy, together with an increased risk of myocardial apoptosis.

Although the role of cardiac autophagy and mitophagy in DOX cardiotoxicity has been controversial for decades (Li et al., 2016; Ma et al., 2017; Wang et al., 2019; Liang et al., 2020), our results kept in line with the notion that DOX overstimulated autophagy and mitophagy, and this overstimulation was harmful to the cardiac physiological activities (Catanzaro et al., 2019). Consistent with previous reports (Wang et al., 2014; Yin et al., 2018), our study revealed that DOX exposure induced autophagy activation, as evidenced by elevated levels of autophagy markers including LC3II, Beclin 1, and ATG5, together with a decreased level of autophagosome–autolysosome fusion marker P62 *in vivo* and *in vitro*. Moreover, transmission electron microscopy measure observed small, round, swollen, and disorganized mitochondrial fragmentations as well as increased autophagic vacuoles engulfing cargos like damaged mitochondria in the hearts of DOX-treated rats. Then, we uncovered that NEU1 inhibitor administration dramatically suppressed DOX-induced autophagy activation and mitochondrial damage. It is well known that mitochondrial damage is an apparent hallmark of DOX cardiotoxicity, since DOX accumulates mainly in mitochondria and nucleus (Wallace et al., 2020). Mitochondrial quality control network would segregate damaged mitochondria or mitochondrial fragmentations by mitochondrial fission for autophagic removal, aiming at maintenance of a functional mitochondrial network. However, excessive mitochondrial fission and mitophagy can form a catastrophic feedback loop, disturbing mitochondrial quality control network and resulting in cell death ultimately (Catanzaro et al., 2019). Previous studies have reported that highly expressed Drp1 and Drp1-dependent excessive mitochondrial fission contributed to DOX-induced cardiomyopathy (Xia et al., 2017; Liang et al., 2020; Zhuang et al., 2021), and Drp1 heterozygous knockout mice were protected against DOX-induced cardiotoxicity (Catanzaro et al., 2019), strongly supporting the critical role of Drp1-dependent mitochondrial fission in DOX-related cardiotoxicity. Our results observed highly expressed Drp1 in the hearts of DOX-treated rats; what is more, the expression of Drp1 was demonstrated to increase in a time-dependent manner under continuous DOX insult in H9C2 cells. Given that mitochondrial fission occurs in coordination with mitophagy, we then explored whether and how mitophagy contributes to DOX-stimulated cardiotoxicity.

PINK1/Parkin-mediated mitophagy is the most extensively studied pathway in autophagic elimination of defective mitochondria (Ashrafi and Schwarz, 2013). Under basal conditions, the level of PINK1 is very low since it is imported to the inner mitochondrial membrane for quick degradation by several proteases; however, damaged mitochondria failed to transport and degrade PINK1, thus resulting in accumulation and stabilization of PINK1 on the outer membrane of mitochondria, which subsequently recruited and activated E3 ubiquitin ligase Parkin, leading to Parkin translocation from cytosol to mitochondrial surface. Activated Parkin leads to ubiquitination of mitochondrial substrates readying for autophagic removal (Pickles et al., 2018). However, the role of PINK1/Parkin-mediated mitophagy in DOX-induced cardiomyopathy is still in dispute. Some studies claimed that enhanced mitophagy could protect against DOX cardiotoxicity (Mancilla et al., 2020; Xiao et al., 2020), while others stood on the opposite (Gharanei et al., 2013; Catanzaro et al., 2019). These mixed results might be explained by the different model used, different DOX concentrations, different time of treatment, and different degree of cell damage. Our results showed that mitophagy-related critical proteins including Parkin and PINK1 were notably increased in parallel with the increased expression of Drp1 in the hearts of DOX-treated rats. Whereafter, *in vitro* studies further showed the accumulation of PINK1 on the mitochondria and the translocation of Parkin from cytosol to mitochondria, together with the ubiquitination of mitochondrial substrates in DOX-treated H9C2 cells, confirming the activation of PINK1/Parkin signaling pathway-regulated mitophagy in DOX-induced cardiomyopathy. Then, NEU1 inhibitor administration not only suppressed the increased expression of Drp1 but also reduced PINK1 accumulation on mitochondria and Parkin translocation to mitochondria in DOX-treated hearts as well as in H9C2 cells. Interestingly, Drp1 inhibitor Mdivi-1 exhibited a synergistic effect with NEU1 inhibitor OSE, strongly indicating that NEU1 inhibitor could attenuate DOX-induced cardiotoxicity through suppressing Drp1-dependent mitochondrial fission and mitophagy.

On the other hand, mitochondrial fission could lead to pro-apoptotic protein release from mitochondria to cytosol, triggering apoptosis and finally cell death (Wan et al., 2018). Emerging evidence also demonstrates that mitophagy undergoes abundant crosstalk with apoptosis (Ham et al., 2020). The activation of mitochondrial-dependent apoptotic pathway in DOX-treated cardiomyopathy has been reported by several groups (Hu et al., 2019; Zhang et al., 2020). In line with these studies, our results revealed an increased myocardial apoptosis caused by DOX both *in vivo* and *in vitro*, whereas NEU1 inhibitor OSE and Drp1 inhibitor Mdivi-1 treatment dramatically improved the apoptotic activities, as evidenced by the decreased expression of pro-apoptotic proteins Bax and Bad, the increased expression of anti-apoptotic protein Bcl-2, and the decreased expression of cleaved caspase-3 and cleaved caspase-9. Therefore, we hold the belief that NEU1 inhibitor could suppress myocardial apoptosis through inhibiting Drp1-mediated excessive mitochondrial fission and mitophagy, subsequently delaying the development of DOX-induced cardiomyopathy.

However, there are some limitations existing in this work. First, further investigations are required to clarify how NEU1 was elevated in DOX cardiotoxicity. Second, the mechanisms of how elevated NEU1 induced Drp1 upregulation remain to be further precisely illuminated. Third, although the cardio-protective effect of NEU1 inhibitor against DOX-induced cardiomyopathy was remarkable in animal models, a large number of clinical trials are needed to confirm the efficacy of existing anti-viral drugs like OSE in DOX-induced cardiomyopathy treatment.

## 5 Conclusion

In conclusion, our study confirmed NEU1 as a crucial inducer of DOX cardiotoxicity by enhancing mitochondrial fission and mitophagy, and inhibition of NEU1 could dramatically improve cardiac dysfunction caused by DOX insult, strongly suggesting the clinical potential of targeting NEU1 as a novel strategy for cardiomyopathy treatment.

## Data Availability

The original contributions presented in the study are included in the article/Supplementary Material, further inquiries can be directed to the corresponding authors.
