# Supplementary material for: Neuraminidase1 Inhibitor Protects Against Doxorubicin-Induced Cardiotoxicity via Suppressing Drp1-Dependent Mitophagy
Source: Front Cell Dev Biol. 2021 Dec 17;9:802502. doi: 10.3389/fcell.2021.802502 (PMC8719652; doi:10.3389/fcell.2021.802502)
Supplement: Supplementary file 1 [file Table1.DOCX]

**Supplementary Table 1.** Primary and secondary antibodies used in this study

| Antibody | Customer | Product number | Dilution |
| --- | --- | --- | --- |
| Neuraminidase1 | Santa Cruz Biotechnology | Sc-166824 | 1:500 |
| LC3A/B | Cell Signaling Technology | 12741 | 1:1000 |
| Becline-1 | Cell Signaling Technology | 3738 | 1:1000 |
| ATG5 | ABclonal Technology | A0203 | 1:1000 |
| P62 | Cell Signaling Technology | 23214 | 1:1000 |
| Drp1 | Proteintech Group | 12957-1-AP | 1:1000 |
| PINK1 | Proteintech Group | 23274-1-AP | 1:1000 |
| Parkin | Proteintech Group | 14060-1-AP | 1:1000 |
| Ubiquitin | Cell Signaling Technology | 58395 | 1:1000 |
| Cleaved caspase 3 | Cell Signaling Technology | 9661 | 1:1000 |
| Cleaved caspase 9 | Cell Signaling Technology | 20750 | 1:1000 |
| Bax | Cell Signaling Technology | 2772 | 1:1000 |
| Bad | ABclonal Technology | A1593 | 1:1000 |
| Bcl2 | Cell Signaling Technology | 3498 | 1:1000 |
| GAPDH | ABclonal Technology | AC002 | 1:10000 |
| VDAC1 | Servicebio | GB111939 | 1:1000 |
| Peroxidase-conjugated AffiniPure Goat Anti-Rabbit IgG(H+L) | JacksonImmunoResearch | 111-035-003 | 1:5000 |
| Peroxidase-conjugated AffiniPure Goat Anti-Mouse IgG(H+L) | JacksonImmunoResearch | 115-005-003 | 1:5000 |
| FITC conjugated Goat Anti-Rabbit IgG(H+L) | Servicebio | GB22303 | 1:200 |
| Cy3 conjugated Goat Anti-Mouse IgG(H+L) | Servicebio | GB21301 | 1:200 |
| Cy3 conjugated Goat Anti-Rabbit IgG(H+L) | Servicebio | GB21303 | 1:200 |
