# Supplementary figures and images for: Neuraminidase1 Inhibitor Protects Against Doxorubicin-Induced Cardiotoxicity via Suppressing Drp1-Dependent Mitophagy
Source: Front Cell Dev Biol. 2021 Dec 17;9:802502. doi: 10.3389/fcell.2021.802502 (PMC8719652; doi:10.3389/fcell.2021.802502)

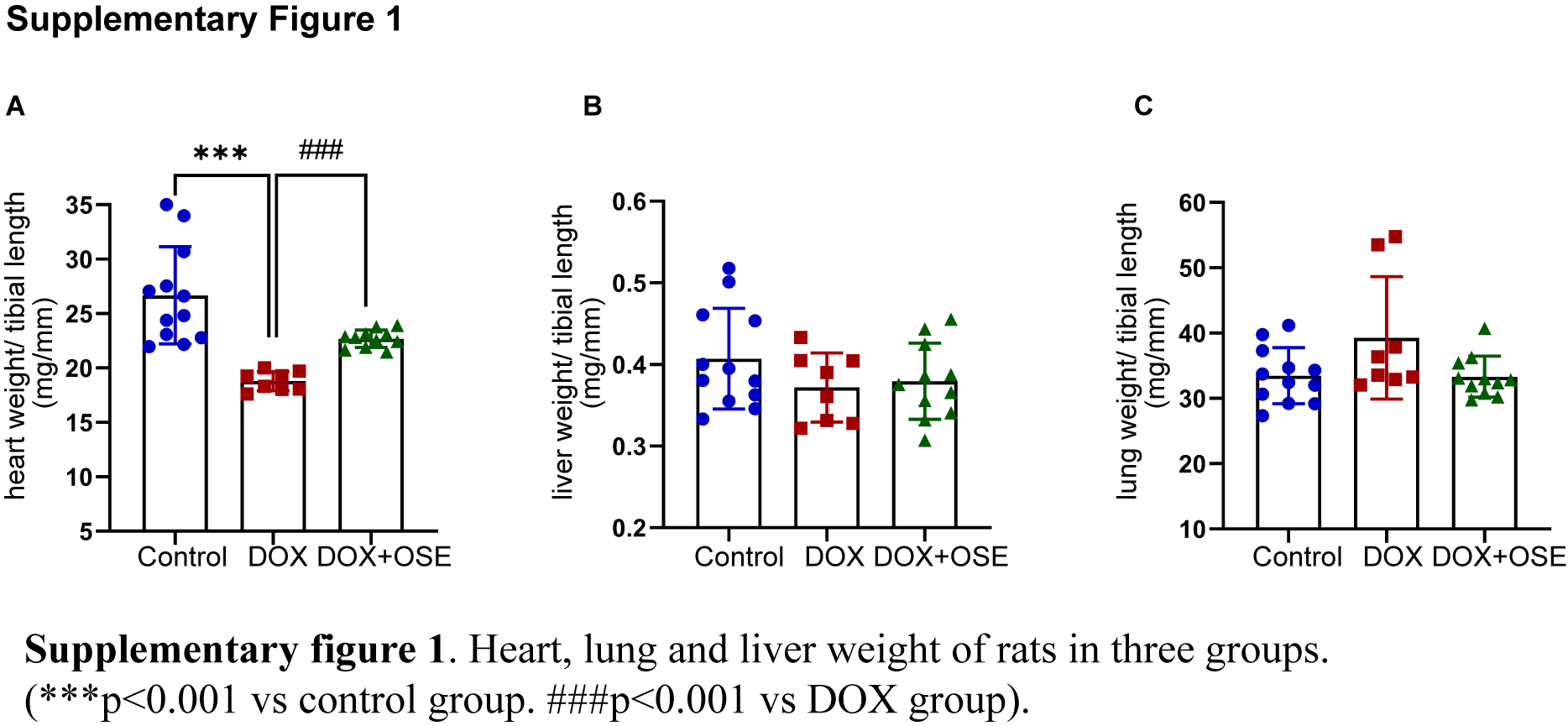

Supplement: Supplementary file 2 [file Image1.TIF]
